# A Network Biology Approach to Denitrification in *Pseudomonas aeruginosa*

**DOI:** 10.1371/journal.pone.0118235

**Published:** 2015-02-23

**Authors:** Seda Arat, George S. Bullerjahn, Reinhard Laubenbacher

**Affiliations:** 1 Department of Mathematics, Virginia Tech, Blacksburg, VA, USA; 2 Virginia Bioinformatics Institute, Virginia Tech, Blacksburg, VA, USA; 3 Department of Biological Sciences, Bowling Green State University, Bowling Green, OH, USA; 4 Center for Quantitative Medicine, University of Connecticut Health Center, Farmington, CT, USA; 5 Jackson Laboratory for Genomic Medicine, Farmington, CT, USA; University of Illinois at Urbana-Champaign, UNITED STATES

## Abstract

*Pseudomonas aeruginosa* is a metabolically flexible member of the Gammaproteobacteria. Under anaerobic conditions and the presence of nitrate, *P. aeruginosa* can perform (complete) denitrification, a respiratory process of dissimilatory nitrate reduction to nitrogen gas via nitrite (*NO*_2_), nitric oxide (*NO*) and nitrous oxide (*N*_2_ *O*). This study focuses on understanding the influence of environmental conditions on bacterial denitrification performance, using a mathematical model of a metabolic network in *P. aeruginosa*. To our knowledge, this is the *first* mathematical model of denitrification for this bacterium. Analysis of the long-term behavior of the network under changing concentration levels of oxygen (*O*_2_), nitrate (*NO*_3_), and phosphate (*PO*_4_) suggests that *PO*_4_ concentration strongly affects denitrification performance. The model provides three predictions on denitrification activity of *P. aeruginosa* under various environmental conditions, and these predictions are either experimentally validated or supported by pertinent biological literature. One motivation for this study is to capture the effect of *PO*_4_ on a denitrification metabolic network of *P. aeruginosa* in order to shed light on mechanisms for greenhouse gas *N*_2_ *O* accumulation during seasonal oxygen depletion in aquatic environments such as Lake Erie (Laurentian Great Lakes, USA). Simulating the microbial production of greenhouse gases in anaerobic aquatic systems such as Lake Erie allows a deeper understanding of the contributing environmental effects that will inform studies on, and remediation strategies for, other hypoxic sites worldwide.

## Introduction

Denitrification is a facultative anaerobic process in which nitrate is utilized as an alternative terminal electron receptor and dissimilatory nitrate is reduced to nitrogen gas via nitrogen oxides [1–3].

$$\begin{array}{c} NO_{3}\overset{}{\to }NO_{2}\overset{}{\to }NO\overset{}{\to }N_{2}O\overset{}{\to }N_{2} \end{array}$$

Since denitrification is one of the few pathways for producing atmospheric *N*_2_, it is a major component of the nitrogen cycle [4]. Denitrification occurs in several habitats such as soils, lakes, rivers and oceans [5]. Nitrogen fluxes from marine systems to the atmosphere are between 25 × 10^9^ and 179 × 10^9^ kilograms per year via microbial denitrification [6]. *Pseudomonas aeruginosa*, a facultative ubiquitous, and metabolically flexible member of the Gammaproteobacteria, can perform (complete) denitrification under anaerobic conditions and the presence of nitrate. Complete denitrification consists of four sequential steps to reduce nitrate (*NO*_3_) to dinitrogen (*N*_2_) via nitrite (*NO*_2_), nitric oxide (*NO*), and nitrous oxide (*N*_2_ *O*), and each step of the pathway is catalyzed by (denitrification) enzymes such as nitrate reductase (nar), nitrite reductase (nir), nitric oxide reductase (nor), and nitrous oxide reductase (nos). The identification and transcriptional control of denitrification genes encoding *nar*, *nir*, *nor* and *nos* has been largely established. Transcription is dependent on a hierarchy of the FNR-like Crp family transcription factors Anr and Dnr, the two-component system NarXL, and the CbbQ family protein NirQ [7, 8], summarized in [4], allowing for experimental validation of *N*_2_ *O* yield as environmental parameters change.

**Fig 1:**
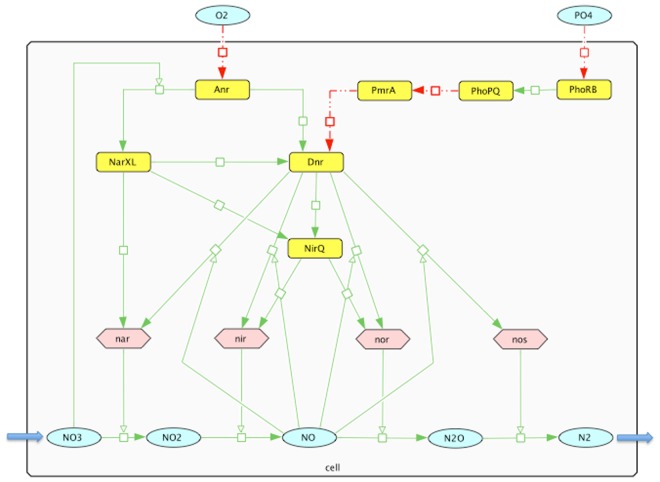
Denitrification regulatory network of *P. aeruginosa*. Green solid arrows indicate upregulation and red dashed arrows indicate downregulation. Model components are PhoPQ, PmrA, Anr, NarXL, Dnr, NirQ, *nar*, *nir*, *nor*, *nos*, *NO*_2_, *NO*, *N*_2_ *O*, and *N*_2_. Our interest lies in perturbation of the external parameters (*O*_2_, *PO*_4_, *NO*_3_) and their effect on the long-term behavior of the network.

We have built a combined gene regulatory and metabolic network for the denitrification pathway in *Pseudomonas aeruginosa* PAO1, a well-studied denitrifier strain (Fig. 1). With this study, we hope to shed light on the environmental factors contributing to greenhouse gas *N*_2_ *O* accumulation, of particular interest in Lake Erie (Laurentian Great Lakes, USA). Environments such as Lake Erie experience seasonal periods of hypoxic conditions favorable for denitrification, and the endemic microbial community regulates expression of alternative respiratory pathways to adapt to low oxygen (*O*_2_) tension.

We are interested in using the model to investigate the effect of *PO*_4_ on the denitrification performance of *P. aeruginosa* under anaerobic conditions with high *NO*_3_. Although there are several studies on regulation of denitrification by kinetic mathematical modeling approaches (e.g. [9–12]), these attempts are not enough to cover the phenomenon at different levels [2]. One of the challenges in building kinetic mathematical models of networks, such as systems of differential equations, is that many of the needed parameters are either not known or unmeasurable. Furthermore, for large networks, kinetic models are difficult to analyze mathematically. Therefore, we take a qualitative approach to model denitrification distinct from the quantitative denitrification models attempted previously. We use a discrete model framework that provides coarse-grained information about the temporal biochemical output of the network in response to environmental conditions. This framework captures attractors (and their biological correspondence, phenotypes) yet it does not render any measurements of time or concentration. In particular, we prefer a time-discrete and multi-state deterministic framework, Polynomial Dynamical System (PDS) [13], to model our denitrification network in *Pseudomonas aeruginosa*.

## Results

The denitrification network consists of molecules, proteins and genes all of which can play an important role in the denitrification process in *Pseudomonas aeruginosa*. Fig. 1 illustrates a static representation of the variables and their regulations. The blue circular nodes are molecules (*O*_2_, *PO*_4_, *NO*_3_, *NO*_2_, *NO*, *N*_2_ *O*, *N*_2_), the yellow rectangular nodes are proteins (PhoRB, PhoPQ, PmrA, Anr, Dnr, NarXL, NirQ) and the pink hexagonal nodes are genes (*nar*, *nir*, *nor*, *nos*) in the network. The large gray rectangle represents the bacterial cell. The regulatory edges between the nodes are either upregulation/activation (green solid arrows) or downregulation/inhibition (red dashed arrows). The pathway begins with the phosphate-sensing two component regulatory system PhoRB [14]. PhoRB, the main *PO*_4_ sensor activating the pho regulon, has been recently shown to be a regulator of PhoPQ transcription in the gammaproteobacterium *Escherichia coli* [15]. In light of the fact that *Pseudomonas aeruginosa* possesses a similar regulatory system to PhoRB in E. coli [16], it is appropriate to label the *PO*_4_-sensing regulatory protein as PhoRB in the denitrification network. In this case, the red dashed arrow from *PO*_4_ to PhoRB means that the availability of phosphate, *PO*_4_, reduces PhoRB function, and the green arrow from PhoRB to PhoPQ means that PhoPQ is activated by PhoRB. Thus, the availability of *PO*_4_ downregulates PhoPQ via PhoRB. The green solid arrow from Anr (anaerobic regulation of arginine deiminase and nitrate reduction) to NarXL and the green solid arrow from *NO*_3_ to the arrow between Anr and NarXL indicate that Anr activates NarXL in the presence of *NO*_3_. In the same setting, PhoPQ inhibits the expression of PmrA [17]. Low oxygen (*O*_2_) tension, which is the major initial signal to turn on the denitrification pathway, can be sensed by Anr [1]. Under anaerobic conditions, Anr primarily promotes Dnr (dissimilatory nitrate respiration regulator) transcription [4]. The effect of Anr on Dnr can be amplified by NarXL [8]. The mechanism of inhibitory effect of PmrA on Dnr [17] is not known, so we assumed that the effect of Anr on Dnr can be reduced by PmrA. The regulatory protein NirQ, which can be activated by NarXL or Dnr, regulates *nir* and *nor* coordinately to keep the level of *NO* low because of toxicity of *NO* [4]. A *NO*_3_-responding regulatory protein, NarXL, directly activates *nar*, and indirectly activates *nir* and *nor* via NirQ [4]. The main regulator of the system, Dnr, controls the expression of all denitrification genes (*nar*, *nir*, *nor*, *nos*) in the presence of *NO* [18]. Of particular note is the influence of the two-component system PhoPQ on PmrA expression and, subsequently, Dnr expression [17], suggesting that phosphorus (P) availability influences denitrification gene expression (see Fig. 1). This is particularly relevant, since linkages between anaerobic Fe(III) reduction and P release adsorbed to *FeOOH* in sediments have been recognized for many years [19, 20], and recently documented in Lake Erie by stable isotope methods [21].

The actual mechanisms of the relationships in the denitrification network (Fig. 1) may be quite complex and involve several intermediates. Thus, the network does not represent a biochemical reaction network, for instance, but rather captures the regulatory logic driving the network in a similar way that a circuit diagram explains the function of a circuit board. In the network (Fig. 1), *O*_2_, *PO*_4_ and *NO*_3_ are *external parameters* and the remaining nodes are *variables*. In the discrete setting that is used to model the denitrification network, each node (e.g. an external parameter *O*_2_ or a variable *nos*) can take up to three states (low, medium, high), and time is implicit and progresses in discrete steps. Our interest lies in perturbation of the external parameters and their effect on the long-term behavior of the variables in the system. S1 Table indicates the discretization values (low/medium/high) for external parameters and nitrogen oxides. Such values incorporate appropriate ranges of long-term nutrient and seasonal oxygen concentrations for Lake Erie [22, 23].

**Fig 2:**
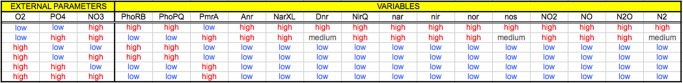
Steady states of the denitrification network under different environmental conditions. The first condition (low *O*_2_, low *PO*_4_ and high *NO*_3_) corresponds to the perfect condition for denitrification and the second condition (low *O*_2_, high *PO*_4_ and high *NO*_3_) corresponds to the denitrification condition disrupted by *PO*_4_ availability. The remaining conditions can be labeled as aerobic conditions.

The denitrification network is an open system; it exchanges molecules with the outside environment and responds to external stimuli [24]. The molecule *NO*_3_ enters the bacterium and *N*_2_ exits the system once the system is triggered by low *O*_2_. The model predicts the long-term behavior of the denitrification pathway under various environmental conditions and these predictions are either supported by the literature or validated by experimental results. Fig. 2 indicates the (predicted) attractors of the system under some possible configuration of the external parameters. There are two conditions that we did not focus on. The low *NO*_3_ and low *PO*_4_ condition and the low *NO*_3_ and high *PO*_4_ condition, while possible, are less likely in freshwaters based on a worldwide survey of lakes revealing that N:P stoichiometric ratios average above the ideal Redfield ratio of 16 [25]. Besides, these conditions would be less relevant to current conditions in Lake Erie, for example, as current measurements of nitrate concentrations (averaging 14*μ*M) typically exceed the Km (Michaelis-Menten constant) for nitrate-dependent denitrification in Pseudomonas spp. (for more information, see [26, 27]). However, a high *P*, high *NO*_3_ condition can arise in lakes affected by agricultural nutrient inputs and deposition of P in sediments.

- **Prediction 1:** If the concentration levels of *O*_2_ and *PO*_4_ are low, and *NO*_3_ is high, then it is a perfect condition for complete denitrification to *N*_2_. The model suggests that all variables in the network except PmrA are expected to be high and the bacterium reduces *NO*_3_ to *N*_2_ via nitrogen oxides. This prediction is *supported* by the following studies [1, 4, 8]. In this condition, Anr senses low *O*_2_ and activates NarXL in the presence of *NO*_3_ [4]. Since the effect of Anr on Dnr is amplified by NarXL but is not reduced by PmrA under low *PO*_4_ conditions, Dnr is highly expressed [8]. The main regulator of the system, Dnr, promotes activation of all denitrification genes (*nar*, *nir*, *nor*, *nos*), so *NO*_3_ is reduced to *N*_2_ via *NO*_2_, *NO* and *N*_2_ *O* [1].
- **Prediction 2:** If the concentration level of *O*_2_ is low, and *PO*_4_ and *NO*_3_ are high, then the model suggests that all variables except PhoRB-PhoPQ are medium or high. Thus, lower complete denitrification activity to *N*_2_ is expected because the *nar*, *nir* and *nor* levels are high whereas the *nos* level is intermediate. This can cause lower rates of reduction of *N*_2_ *O* to *N*_2_ i.e. higher rates of accumulation of *N*_2_ *O*. These predictions *coincide* with the following studies [8, 17] and experimentation. In this condition, Dnr level is intermediate and induces the expression of denitrification genes (*nar*, *nir*, *nor*, *nos*) due to the fact that the effect of Anr on Dnr is amplified by NarXL and is reduced by PmrA [8, 17]. Moreover, our experimental results in Table 1 show a modest increase in *N*_2_ *O* production with a high *PO*_4_ level. There is about a 2-fold increase in *N*_2_ *O* concentration in comparison of the anaerobic *P. aeruginosa* culture with 1.0*mM* *PO*_4_ to the anaerobic *P. aeruginosa* culture with 7.5*mM* *PO*_4_. Under these conditions, the culture at 1.0*mM* *PO*_4_ is grown under the ideal total N:P ratio of 16 reflecting the 16:1 N:P elemental stoichiometry of aquatic plankton [28]. The cultures grown at elevated *PO*_4_ (3.0*mM* and 7.5*mM*) thus reflect a condition in which *PO*_4_ is available at surplus levels that repress the PhoRB-dependent gene activation. This is an example of how *PO*_4_ can influence the expression of denitrification gene, *nos*, distant from *PO*_4_ acquisition and subsequently greenhouse gas *N*_2_ *O* accumulation.
- **Prediction 3:** If the concentration level of *O*_2_ is high, then, the model suggests that there is no denitrification activity regardless of the values of the other external parameters (*PO*_4_ or *NO*_3_). This prediction is *supported* by Zumft’s extensive review paper, which states that under aerobic conditions, Pseudomonas aeruginosa cannot perform denitrification because Anr cannot activate the main regulator of the system, Dnr, in the presence of oxygen [1].

**Table 1:** Nitrous oxide concentration in *P. aeruginosa* cultures grown in glucose minimal medium at varying phosphate concentrations, normalized to 10^8^ cells.

Culture (mM *PO*_4_)	[*N*_2_ *O*] ppm, 24 h	[*N*_2_ *O*] ppm, 72 h
1.0 mM	760.3 +/− 109.3	813.8 +/− 52.1
3.0 mM	856.0 +/− 121.5	872.3 +/− 63.3
7.5 mM	1484.0 +/− 146.2	1786 +/− 98.0

Fig. 2 indicates the attractors of the system under different environmental condition. These attractors indeed are steady states, each of which corresponds to one environmental condition. This agrees with biology; Palsson highlighted that open systems eventually reach a (homeostatic) steady state and are in balance with their environment until the environmental conditions are perturbed [24]. Phenotypes, biological interpretations of the long-term behavior (steady states), of the system under various environmental conditions can be found in Table 2. Based on the steady state analysis above, the Pseudomonas network model predicts that elevated *PO*_4_, hypothesized to increase under hypoxia, acts to modulate the transcriptional network to limit *nos* gene expression. Thus, the physiological output under this condition will be an increased yield of *N*_2_ *O* relative to *N*_2_. Given the prediction that increased *PO*_4_ will influence the *N*_2_ *O* yield, our experimental results thus far indicate that *PO*_4_ availability modestly, but significantly increases *N*_2_ *O* yield in this model species (ANOVA *p* = 0.012; Table 1). While other studies have suggested linkages between *N*_2_ *O* accumulation and factors such as *nosZ* vs. *nirS/K* abundance [29, 30], *nirS* (heme dependent nitrite reductase) genetic diversity [31], or soil pH [32], the data presented here are the first to suggest a role for *PO*_4_ in regulating the denitrification pathway. Given the elevated *PO*_4_ release from *FeOOH* complexes following sedimentary anaerobic Fe(III) reduction [19, 20], hypoxia may yield a high P, high *NO*_3_ condition that enhances *N*_2_ *O* production.

**Table 2:** Biological interpretation of the steady states of the system under different environmental conditions.

*O*_2_	*PO*_4_	*NO*_3_	BIOLOGICAL INTERPRETATION
low	low	high	high denitrification performance
low	high	high	low denitrification performance
high	low	low	no denitrification
high	low	high	no denitrification
high	high	low	no denitrification
high	high	high	no denitrification

## Discussion

In an aquatic system, oxygen dissolves in water to be available to living aerobic organisms. Hypoxia is the phenomenon of dissolved oxygen below 4*mgO*_2_ per liter. Common reasons for hypoxia include aerobic respiration of decaying algal biomass from bloom events. Such blooms are fueled by increased availability of N and P due to anthropogenic inputs such as agricultural runoff and industrial pollutants [33]. The linkage between high nutrient (*N*, *P*) loads and *N* losses (*N*_2_ and *N*_2_ *O*) through dissimilatory anaerobic processes was described recently [34]. Hypoxic (low-oxygen) areas, so-called dead zones, often occur in several large bodies of water affected by human activity, including Lake Erie, which is of particular interest. Establishing a better understanding of the nutrient cycling of Lake Erie has quite wide ranging socioeconomic impacts on its recreational area and economy, primarily fisheries. Through denitrification, dead zones lead to microbial production of the greenhouse gas nitrous oxide (*N*_2_ *O*), which plays a crucial role in ozone layer depletion and climate change. Simulating the microbial production of greenhouse gases in anaerobic aquatic systems such as Lake Erie allows a deeper understanding of the contributing environmental effects that will inform studies on, and remediation strategies for, other hypoxic sites worldwide. During hypoxia, the denitrification rate in Lake Erie is about 150*μ*mol*N*_2_ *m* ^−2^ *h* ^−1^ [35]. In addition to oxygen, the intersections of the nitrogen cycle with other geochemical cycles may be important factors influencing denitrification and nitrogen (N) sinks in aquatic systems. In particular, the increased availability of phosphorus (*P*) has been shown to dictate the rate of nitrogen removal in aquatic systems [34]. Indeed, the transcriptional regulatory network developed for *P. aeruginosa* indicates that bioavailable phosphate (*PO*_4_) is an environmental factor that should be considered.

The bacterium *Pseudomonas aeruginosa* is an example of an abundant microbe in aquatic systems [36], and analysis of Lake Erie metagenomic data sets reveals abundant pseudomonads capable of denitrification (Unpublished data, DOE-JGI). This study describes a computational model of a denitrification network of this bacterium to capture the effect of *PO*_4_ on its denitrification performance in order to shed light on greenhouse gas *N*_2_ *O* accumulation during oxygen depletion. To our knowledge, this is the *first* mathematical model of denitrification for this bacterium. Transcription is dependent on a hierarchy of the FNR-like Crp family transcription factors Anr and Dnr, the two-component system NarXL, and the CbbQ family protein NirQ [7, 8, 37], allowing for experimental measurement of *N*_2_ *O* as external (environmental) parameters change. The model was constructed based on the pertinent biological literature. Model predictions either agree with current published results or are validated by experimentation. The new biology that our model discovers is that *PO*_4_ availability strongly affects the denitrification activity of *P. aeruginosa* under anaerobic conditions and the presence of nitrate; high *PO*_4_ can cause less *N*_2_ *O* reduction to *N*_2_ during denitrification. The data presented here are the first to suggest a role for *PO*_4_ in regulating the denitrification pathway in *Pseudomonas aeruginosa*.

Current efforts will be expanded to determine how *PO*_4_ affects greenhouse gas *N*_2_ *O* accumulation during denitrification in *P. aeruginosa*. According to the model, the activation of Dnr by Anr or the activation of *nos* in the presence of *NO* by Dnr can be prevented by high *PO*_4_. These hypotheses will be tested utilizing quantitative reverse transcriptase PCR (qRT-PCR) to determine Dnr, *norB* (nitric oxide reductase large subunit gene) and *nosZ* (encoding nitrous oxide reductase) transcript levels in denitrifying cultures grown in increasing *P*. Synergistic interactions between individual members of population of *Pseudomonas aeruginosa* may need to be taken into account and incorporated to the model. For instance, Toyofuku and his colleagues stated that denitrification performance of *P. aeruginosa* does not only depend upon activation of denitrification genes (*nar*, *nir*, *nor*, *nos*) but also cell-cell communications under denitrifying conditions [38].

The model described here works well for cultured *Pseudomonas*, and the next step is to test natural complex microbial communities from different denitrification sites. The effects of *PO*_4_ on *N*_2_ *O* production will be tested in mesocosms of hypoxic Lake Erie water samples to see if the model described here predicts the community as a whole. By testing the model on environmental samples in mesocosms from Lake Erie and elsewhere, the study can likely be applied broadly to other marine dead zones such as those that routinely occur in the Gulf of Mexico.

## Materials and Methods

### Computational Methods

Our network consists of two different sub-networks (metabolic and gene regulatory) and consequently different time scales. From a discrete modeling perspective, this issue can be tackled or ignored only if the long-term behavior of the system is of interest. One could address this issue either (1) using a stochastic framework such as Stochastic Discrete Dynamical System (SDDS) [39] if how fast/slow the reactions are in the network are known/inferred out of a time-course experimental data or (2) introducing time delays by an asynchronous update schedule. Due to inadequate information on the reaction rates, we do not focus on a stochastic framework. Even with a fully asynchronous update schedule, the attractors are preserved for each configuration of external parameters; however, this asynchronous update schedule requires more time steps to reach a steady state than a synchronous update schedule does. Since an asynchronous update schedule provides us more on transient behavior of the system and we are interested in long-term behavior of the system, we prefer to use a deterministic framework with a synchronous update schedule, Polynomial Dynamical System (PDS), which allows us to model regulatory networks over a finite field [13].

**Definition 1** *Let x*_1_, *x*_2_, …, *x_n_ be variables which can take values in finite fields X*_1_, *X*_2_, …, *X*_*n*_ *respectively. Let* **X** = *X*_1_ × ⋯ × *X*_*n*_ *be the Cartesian product. For each i* = 1, 2, …, *n, we define f_i_*: **X** → *X_i_ which is an update function that describes the regulation of x_i_ through interaction with other variables in the system. A Polynomial Dynamical System is a collection of n update functions*

$$\begin{array}{c} f=\left(f_{1},f_{2},\;\ldots ,f_{n}\right):X\overset{}{\to }X \end{array}$$

In the model, all external parameters (*O*_2_, *PO*_4_, *NO*_3_) and some variables (PhoPQ, PmrA, Anr, NarXL) are Boolean (low or high), and other variables are ternary (low, medium or high). There are 14 variables, each of which is labeled for the mathematical formulation. Table 3 indicates the variables, their discretization, update rules and the literature evidence that support these update rules. Inflow substances (i.e. external parameters: *O*_2_, *PO*_4_, *NO*_3_) in this model give inputs to variables and are involved in the update rules. They do not have update rules because not only they do not have regulators but also we are interested in analyzing the long-term behavior of the model under different configurations of them. The model has only one outflow substance, *N*_2_, whose regulation depends upon the greenhouse gas *N*_2_ *O* and its reductase, *nos*.

Based on the literature, we formulate the regulation of the variables with MIN, MAX and NOT, which correspond to AND, OR and NOT in a Boolean setting. The following are examples for how the update rules are decided:

**Table 3:** Summary of the model variables, their discretization, update rules and supportive argument. The update rules with an asterix (*) means this update rule is very close to the biological correspondence but not quite. The transition tables of the variables having update rules with an asterix (*) can be found in the Supplementary material.

Index	Variables	Discretization	Update Rules	Literature Evidence
1	PhoRB	Boolean	*NOT*(*PO*_4_)	PhoRB is a phosphate-sensing two component regulatory system [14]
2	PhoPQ	Boolean	*PhoRB*	PhoPQ regulates acid phosphatase under P starvation [48, 49], and PhoB regulates PhoQ transcription [15]
3	PmrA	Boolean	*NOT*(*PhoPQ*)	PhoPQ inhibits the expression of PmrA [17]
4	Anr	Boolean	*NOT*(*O*_2_)	Low oxygen (*O*_2_) tension can be sensed by Anr [1]
5	NarXL	Boolean	*MIN*(*Anr*, *NO*_3_)	Anr activates NarXL in the presence of *NO*_3_ [4]
6	Dnr	Ternary	*MIN(*Anr*, *MAX*(*NarXL*, *NOT*(*PmrA*)))	The effect of Anr on Dnr can be reduced by PmrA (model assumption) and Anr and NarXL cooperatively activate *dnr* [8, 17]
7	NirQ	Ternary	*MAX(*NarXL*, *Dnr*)	NirQ can be activated by NarXL or Dnr [4]
8	*nar*	Ternary	*MIN(*NarXL*, *MIN*(*Dnr*, *NO*))	*nar* is directly activated by NarXL and Dnr in the presence of *NO* [4, 18]
9	*nir*	Ternary	*MAX*(*NirQ*, *MIN*(*Dnr*, *NO*))	*nir* is activated by NirQ or Dnr in the presence of *NO* [4, 18]
10	*nor*	Ternary	*MAX*(*NirQ*, *MIN*(*Dnr*, *NO*))	*nor* is activated by NirQ or Dnr in the presence of *NO* [4, 19]
11	*nos*	Ternary	*MIN*(*Dnr*, *NO*)	*nos* is activated by Dnr in the presence of *NO* [18]
12	*NO*_2_	Ternary	*MIN(*NO*_3_, *nar*)	*NO*_3_ is reduced to *NO*_2_ by *nar* [1]
13	*NO*	Ternary	*MIN*(*NO*_2_, *nir*)	*NO*_2_ is reduced to *NO* by *nir* [1]
14	*N*_2_ *O*	Ternary	*MIN*(*NO*, *nor*)	*NO* is reduced to *N*_2_ *O* by *nor* [1]
15	*N*_2_	Ternary	*MIN*(*N*_2_ *O*, *nos*)	*N*_2_ *O* is reduced to *N*_2_ by *nos* [1]

- An update rule of NarXL can be defined as “MIN (Anr, *NO*_3_)” because NarXL is activated by Anr only in the presence of *NO*_3_, i.e. both Anr and *NO*_3_ need to be high for NarXL regulation.
- An update rule of *nir* can be labeled as “MAX (NirQ, MIN(Dnr, *NO*))” due to the fact that *nir* is activated by NirQ or Dnr in the presence of *NO*.
- An update rule of PhoRB can be “NOT (*PO*_4_)” since *PO*_4_ downregulates PhoRB, i.e. one is low when another is high.

From the update rules in Table 3, for each network variable, we constructed a corresponding transition table, which describes how a specific variable responds to different configurations of their regulators. Although the regulations for most variables can be formulated by MIN, MAX and/or NOT, the regulations of a few variables are very close to some formulation but not quite. For the sake of consistency with biology, we decided to slightly modify the transition table of Dnr, NirQ, *nar* and *NO*_2_, whose update rules are marked with an asterix (*) in Table 3. The transition tables of these variables and more explanation on why the changes were necessary can be found in S2 Table, S3 Table, S4 Table and S5 Table respectively.

Besides, if the variable takes three states (low, medium, high), the current state of the variable is included its own transition table. This does not mean autoregulation/self-regulation; but it is to prevent the variable from jumps between the low (0) state and the high (2) state at the next time step. In other words, including the current state of a ternary variable in its transition table provides a smooth transition among its own states. On the other hand, such jumps cannot occur in a Boolean variable.

After constructing a transition table for each variable *x*_*i*_, an update function can be obtained by interpolating its transition table using the polynomial form:

$$\begin{array}{c} f_{i}\left(x\right)=\sum_{\left(c_{i_{1}},\ldots ,c_{i_{r}}\right)\in F_{p}^{r}}f_{i}\left(c_{i_{1}},\ldots ,c_{i_{r}}\right)\prod_{j\in \{i_{1},\ldots ,i_{r}\}}\left(1-{\left(x_{j}-c_{j}\right)}^{p-1}\right)\;\left(\mathrm{mod}\;p\right) \end{array}$$ (1)

where **x** = (*x*_*i*_1__, …, *x*_*i*_*r*__) is a vector; *c*_*i*_1__, …, *c*_*i*_*r*__ are the values of the variables *x*_*i*_1__, …, *x*_*i*_*r*__, which affect the update of *x*_*i*_ in the transition table of *x*_*i*_; *f*_*i*_(*c*_*i*_1__, …, *c*_*i*_*r*__) is the value in the last column of the transition table of *x*_*i*_; *p* is the maximum (prime) number of the different discrete values that all variables can take on [40]. In our model, all computations were done in modulo 3.

After having all update functions (see S1 Text), we computed the basin of attraction of the whole system under the environmental conditions of interest (see Fig. 2). For model construction and steady state analysis, we used customized Ruby and Perl scripts, which are a part of the source code of Analysis of Dynamic Algebraic Models (ADAM, available at http://adam.plantsimlab.org/), a free of charge web-tool to analyze the dynamics of discrete biological systems [41].

### Experimental Methods

*Pseudomonas aeruginosa* PAO1 cultures were grown in stoppered 20*mL* serum vials containing glucose minimal medium [42] supplemented with 110*mM* glucose and 16*mM* nitrate (*NO*_3_). Phosphate (*PO*_4_) concentration varied from 1.0*mM* to 7.5*mM*, and triplicate culture vials were sampled for headspace gases at 24*h* and 72*h* post-inoculation. Gases were dispensed into evacuated exetainers and assayed for nitrous oxide by gas chromatography. Gas production was normalized to cell counts obtained by flow cytometry of culture fluids.

## Supporting Information

S1 Table Discretization of external parameters and nitrogen oxides. Information in the table was obtained from [43–47]
(XLS)

S2 Table Transition table of Dnr. (XLS)

S3 Table Transition table of NirQ. (XLS)

S4 Table Transition table of *nar*. (XLS)

S5 Table Transition table of *NO*_2_. (XLS)

S1 Text Update functions of all variables in the denitrification network. (TXT)
